# Supplementary material for: Machine learning-based prediction of metabolic dysfunction-associated steatotic liver disease using National Health and Nutrition Examination Survey (NHANES) data
Source: PLoS One. 2025 Nov 12;20(11):e0335656. doi: 10.1371/journal.pone.0335656 (PMC12611120; doi:10.1371/journal.pone.0335656)
Supplement: Attachment — Submitted filename: Review of Article-20250829.docx [file pone.0335656.s002.docx]

**Summary:**

This study developed a machine learning model to predict non-alcoholic fatty liver disease (NAFLD), a condition affecting about 25% of the global population and linked to cirrhosis and liver cancer. Using data from 2,460 U.S. adults in the 2017–2020 NHANES survey, participants with excessive alcohol use, hepatitis B/C, or incomplete records were excluded. The dataset was split 80:20 into training and testing sets. Five algorithms were compared, with XGBoost performing best. Recursive Feature Elimination identified 12 key predictors, including waist circumference and BMI. The model achieved an AUC of 0.874 on the test set. This efficient and stable tool supports early identification of high-risk individuals and could improve NAFLD prevention and control strategies. However, there are still some comments that should be addressed by the author.

**Comments:**

1. Your current keywords are strong but could be broadened for better indexing. In addition to Non-alcoholic Fatty Liver Disease (NAFLD), Machine learning, XGBoost, and NHANES, you should also include Prediction model and Risk factors to highlight the study’s focus and clinical relevance.
2. The introduction clearly emphasizes the global burden of NAFLD, but could the authors provide more recent prevalence estimates or regional variations? Strengthening the link between NAFLD and systemic diseases is important, but some additional references on cardiovascular outcomes might enhance the context.
3. The rationale for early diagnosis is well-stated, though the authors might elaborate on why NAFLD remains so underdiagnosed despite its prevalence. Could more detail be given on the specific clinical challenges in identifying patients at the simple steatosis stage?
4. The authors correctly highlight the potential of machine learning, but have they considered why prior models underperformed in clinical translation? More detail on how this study differs from earlier work (e.g., larger dataset, feature optimization) would clarify its novelty.
5. The stated aim is clear, but could the authors explain how RFE specifically addresses the limitations of earlier models? Emphasizing the potential clinical utility of the model would make the objective more compelling for a broad readership.
6. The use of NHANES is appropriate given its representativeness, but could the authors clarify whether sampling weights were applied to account for the complex survey design? Additionally, the exclusion of participants with missing data via case-wise deletion may reduce sample size and introduce bias—have the authors considered imputation methods as a sensitivity analysis?
7. The demographic, physical, and biochemical variables are well-justified, but are there additional metabolic or inflammatory markers in NHANES (e.g., triglycerides, HDL, fasting glucose) that could enhance prediction accuracy? It would also be helpful if the authors explained why certain common NAFLD predictors were excluded, and whether multicollinearity among features (e.g., BMI and waist circumference) was assessed.
